# Necrotizing Fasciitis Occurring After a Conventional Tattoo

**DOI:** 10.7759/cureus.55368

**Published:** 2024-03-01

**Authors:** Malvine Vogel, Jonathan De Bodt, Jean-Marie Jacques

**Affiliations:** 1 Emergency Department, Epicura Hospitals, Hornu, BEL

**Keywords:** broad-spectrum antibiotic, fasciotomy, sepsis, tattooing, necrotizing fasciitis

## Abstract

A 34-year-old patient presented to the emergency department after getting a tattoo from a licensed tattoo artist at home. The patient was in septic shock with renal failure, and the clinical examination revealed a skin infection at the tattoo site. Suspecting necrotizing fasciitis, the patient underwent fasciotomy with deep tissue sampling and vacuum-assisted closure therapy. Broad-spectrum antibiotic therapy was initiated and later adjusted based on bacterial culture results. The patient quickly recovered and was discharged from the intensive care unit.

We report the first case of post-conventional tattoo necrotizing fasciitis in Belgium. The tattoo was performed by a professional licensed tattoo artist, equipped as required by Western legislation. Previously reported cases highlighted necrotizing fasciitis occurring after Samoan or Samoan-style tattoos, an ancestral practice with handmade tools. In our case, the tattoo was done in a conventional way with modern tools and techniques. It is the lack of hygiene precautions that we can attribute to the development of this serious pathology.

## Introduction

Tattoos are increasingly popular practices in Western countries, with an estimated 10 to 20% of the population currently tattooed [1]. Several complications are associated with this practice, ranging from benign (eczema, lymphadenopathy, local edema) to severe, including some reported cases of necrotizing fasciitis [1-2]. These complications are typically linked to practices with poor hygiene or ancestral manual tattoo techniques [2-3].

Necrotizing fasciitis is a rare, rapidly progressive soft tissue infection that causes severe illness and, in some cases, death. Most patients who develop necrotizing fasciitis have pre-existing conditions such as diabetes, chronic liver disease, or renal disease. In rare cases, it can occur in young and healthy patients [3].

In most cases, this pathology occurs in the presence of a wound or injury. These are usually operation sites, burns, and ulcers, but more recently, piercings or tattoos have also been reported as causes of necrotizing fasciitis. This severe complication of tattoos has been mainly described in a few cases related to traditional Samoan tattoos, an ancestral tattooing technique using handmade tools, which is not a well-regulated practice [1-3].

To date, only one case in the literature reports the occurrence of necrotizing fasciitis with fasciotomy after a tattoo with modern equipment and adherence to hygiene rules in a young and healthy patient [4]. We report the occurrence of a second case of this nature.

## Case presentation

A 34-year-old patient presented to the emergency department with a fever. On arrival, the patient was hypotensive (blood pressure: 80/40 mmHg), tachycardic (heart rate: 120 bpm), and had a peripheral temperature of 39.5°C. The patient appeared pale and lethargic.

**Figure 1:**
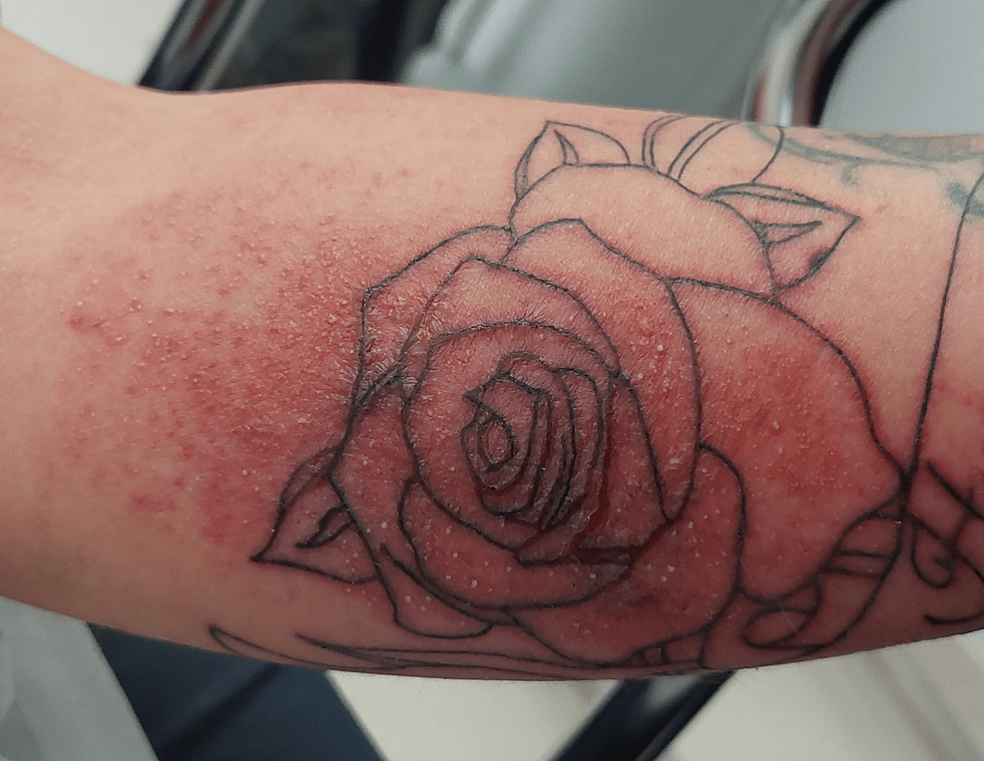
Erythema and edema of the patient's forearm at the new tattoo site

The patient had no medical history and wasn’t on any chronic medication. Four days prior, the patient had a tattoo done by a licensed professional using his regular electric equipment, but at the patient's home. The professional was the patient's brother-in-law, who came to tattoo him at the patient's request. The tattoo was in the middle of the right forearm. Clinical examination revealed local edema with erythema at the tattoo site, and an unpleasant odor was noted upon removal of the protective dressing (Figure 1).

Laboratory parameters showed an inflammatory syndrome with a C-reactive protein of 356 mg/L (reference: < 5 mg/L), an acute kidney injury classified at stage three (creatinine: 3.71 mg/dL, glomerular filtration rate (GFR): 18.8 mL/min/1.73 m^2^, reference creatinine: 0.66-1.25 mg/dL, reference GFR: > 60 mL/min/1.73 m^2^), and hyperlactatemia at 3.21 mmol/L (reference: < 2 mmol/L). Liver function and electrolytes were within normal limits.

**Figure 2:**
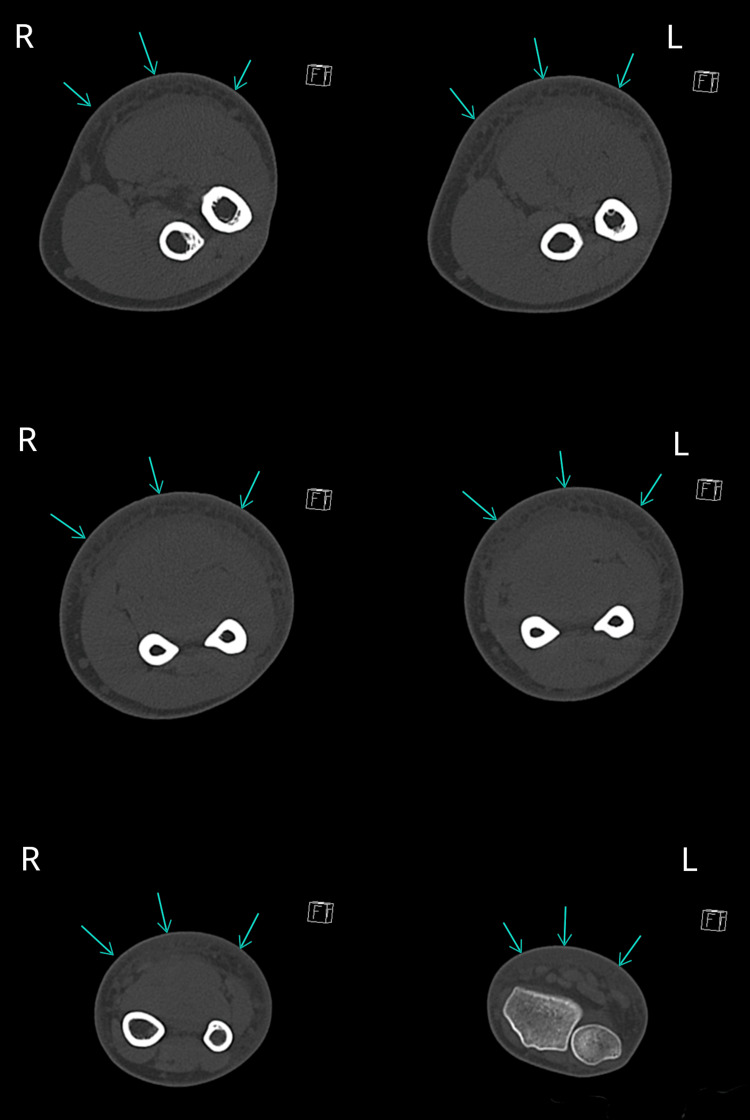
Axial CT of the forearm shows subcutaneous fat infiltration and collection along the muscle planes from elbow to wrist CT: computed tomography

**Figure 3:**
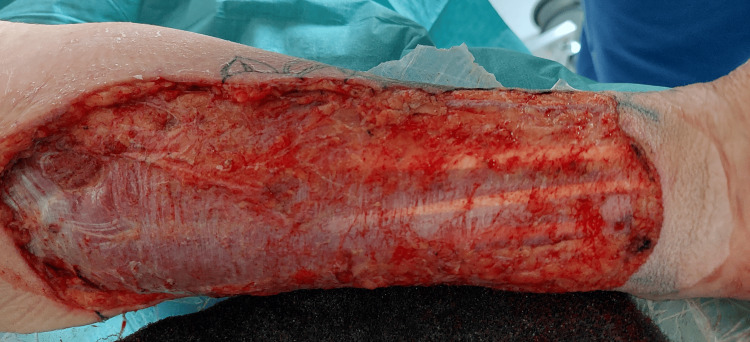
Fasciotomy wound post-debridement of the forearm

A forearm CT scan revealed subcutaneous fat infiltration with a deep collection along the muscle planes throughout the anterior aspect of the forearm, from wrist to elbow (Figure 2). We suspected necrotizing fasciitis, which was confirmed during fasciotomy with deep tissue sampling. The fasciotomy was extended to the entire forearm due to the significant spread of necrotizing fasciitis (Figure 3). The fasciotomy site was covered with a vacuum-assisted closure therapy dressing.

The patient was treated with piperacillin, tazobactam, and vancomycin and then transferred to the ICU postoperatively. The first 48 hours in the ICU corrected lactic acidosis and renal failure. The patient did not require inotropic support.

Antibiotic therapy was also adjusted based on deep tissue culture results, revealing methicillin-sensitive *Staphylococcus aureus* and *Enterobacter hormaechei*. Flucloxacillin and clindamycin were initiated, and other antibiotics were discontinued. The patient was transferred to the plastic surgery unit for the completion of antibiotic therapy and an eventual skin graft.

## Discussion

Necrotizing fasciitis is an infection of deep subcutaneous tissues, causing the destruction of muscle fascia and adipose tissue. Its estimated incidence is 0.3 to 15 cases per 100,000 inhabitants [5]. The practice of tattooing dates to ancient times and has become increasingly common in Western countries, where 10 to 20% of the population is currently tattooed [1]. The tattooing process involves injecting pigments into subcutaneous tissues with a needle, causing an inflammatory reaction and an influx of immune cells [6]. Tattooing can have side effects such as an allergic reaction to pigments or increased skin sensitivity to the sun, leading to burning and skin depigmentation. Approximately 67.5% of tattooed patients report mild side effects such as bleeding, pain, eczema, and local edema [1,4].

The tattooing process involves a breach of the skin barrier, posing a significant risk of infection as the skin is not sterile. Approximately 1-5% of tattooed individuals experience bacterial infections associated with this procedure [2].

Infectious complications following tattoos have been described for years, usually limited and benign [7]. More rarely, cases of necrotizing fasciitis have been reported [1,3,8]. Sepsis caused by necrotizing fasciitis is associated with lactic acidosis and acute renal failure in most cases, as in our patient [4,9]. The mortality of necrotizing fasciitis is estimated between 25 and 35%, making it a morbid condition when not diagnosed promptly [5].

Necrotizing fasciitis is a life-threatening emergency, with treatment consisting of fasciotomy and broad-spectrum intravenous antibiotic therapy [4]. ICU monitoring is standard, and in the long term, surviving patients require reconstructive surgery and skin grafts [5].

We searched for literature in MEDLINE (PubMed) to collect case reports about necrotizing fasciitis following tattooing. The prevalence of this condition in tattooed populations is unknown, with only seven cases reported in the literature. These cases have all been associated with traditional Samoan tattooing methods in Oceania [1,3,4,10]. Traditional Samoan tattooing is an ancestral technique common in Polynesia and New Zealand. This tattoo is created by handmade tools that are boiled in water before use. Samoan tattoos take several months to be done [11]. According to Das et al., Dieckmann et al., and Elegino-Steffens et al., the traditional tattooing method and cases of associated necrotizing fasciitis can be explained by hygiene deficiencies in this technique, especially the lack of sterilization [3,8,10].

Such complications are rare in Western countries due to hygiene regulations (regarding sterilization, workspace, and equipment) governing tattoos and those who own a tattoo studio. There are standard protocols established by law that guarantee the highest level of safety possible. Only one case reports the occurrence of necrotizing fasciitis after a tattoo with modern equipment and adherence to hygiene rules. The tattoo reported in this case was made in Samoan style with modern equipment [4]. In our case, the tattoo was more standard style. Although the equipment met hygiene standards and was used by a licensed professional, the tattoo was done at the patient's home with an electric machine, suggesting that usual hygiene precautions were not taken.

The contamination could be explained by several phenomena: the ink used may have been contaminated due to the prior opening of the bottle; the needle may not have been properly disinfected or reused without asepsis; the disinfection of the tattoo area was lacking; or the patient may have scratched the recent tattoo, thereby introducing germs [12]. In this case, it is the obvious lack of hygiene and the execution of this tattoo without usual precautions in a dedicated environment that is at the origin of the necrotizing fasciitis. Best practice guidelines were not followed, both in terms of disinfection and hygiene precautions, as well as in the recommendations for the use of equipment.

## Conclusions

Tattoos are increasingly popular practices worldwide, sometimes associated with rare cases of necrotizing fasciitis. This challenging pathology has a high mortality rate. To our knowledge, only eight cases of necrotizing fasciitis after tattooing were published. Seven of them are consequences of traditional Samoan tattooing, an ancient Oceanian practice that relates to a lack of hygiene. This is only the second reported case in Western countries occurring after a tattoo made by a professional tattooist, diagnosed in a timely manner, and treated with fasciotomy and broad-spectrum antibiotic therapy, allowing rapid discharge from intensive care. Our clinical case involved necrotizing fasciitis at a tattoo site from a tattoo done at home, likely under inadequate hygiene conditions.

Tattooing techniques used in Western countries, even with strict and regulated hygiene practices, can pose significant health risks to patients. The most severe complication is necrotizing fasciitis, a life-threatening condition that we wanted to bring to the attention of clinicians.
